# Rhinophyma

**DOI:** 10.11604/pamj.2017.26.122.11240

**Published:** 2017-03-03

**Authors:** El Mahi Hakima, Mernissi Fatima Zahra

**Affiliations:** 1 Department of Dermatology, University Center Hassan II, Fez, Morocco

**Keywords:** Rhinophyma, surgical treatment, decortication

## Image in medicine

Rhinophyma (rhis: nose, phyma: growth) is a rare, disfiguring disease characterized by a progressive hypertrophy of the soft-tissues of the nose with its increased volume, mainly in the lower half, and often associated to an end-stage of severe acne rosacea. The cause of rhinophyma is unknown, but it is believed to be the end stage of acne rosacea. The diagnosis is easy to ascertain based on the clinical features of the disease. In advanced cases, medical management is believed to be inferior to the results seen with surgical treatment. Surgical management is divided into 3 approaches: full-thickness excision followed by split-thickness skin graft, full-thickness excision followed by full-thickness skin graft, and decortication or partial excision. Decortication techniques include dermaplaning, dermabrasion, cryosurgery, electrosurgery, laser surgery and sharp blade excision. We reporte a case treated by a decortication technique: a 60-year-old man who presented at the dermatology department with a 20-year history of rhinophyma. He had never undergone any laser or surgical interventions. No other medical problem was reported, and there was no family history of rosacea. In collaboration with the Department of Plastic Surgery, rhinophymectomy using a scalpel and electroscalpel was undertaken. The procedure was performed with the patient under general anesthesia. A scalpel with a no. 15 blade was used. The postoperative period was satisfactory. The first signs of skin reepithelialization appeared 2 weeks after the intervention. At a 2 month-follow-up visit, a scar was present on the nasal tip, but the overall nasal contour was restored.

**Figure 1:**
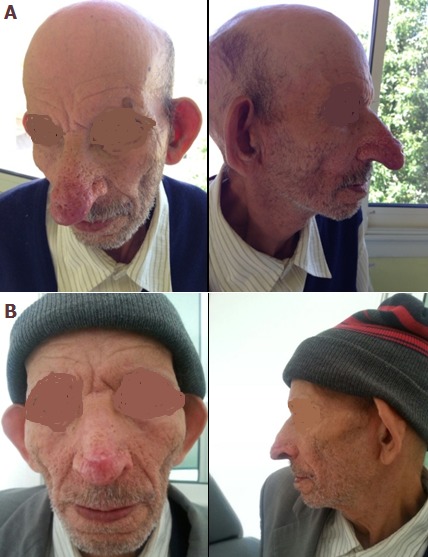
(A) preoperative view; (B) view 8 weeks postoperatively

